# Comparison of Long-Term Outcomes of Monotherapy and Polytherapy in Seizure-Free Patients With Epilepsy Following Antiseizure Medication Withdrawal

**DOI:** 10.3389/fneur.2021.669703

**Published:** 2021-05-24

**Authors:** Yuxuan Wang, Li Xia, Rong Li, Yudan Li, Jingyi Li, Qin Zhou, Songqing Pan

**Affiliations:** Department of Neurology, Renmin Hospital of Wuhan University, Wuhan, China

**Keywords:** antiseizure medication withdrawal, seizure relapse, monotherapy, polytherapy, risk factor

## Abstract

**Objective:** The objectives of this study were to compare the risk and timing of seizure relapse in seizure-free patients with epilepsy following the withdrawal of monotherapy or polytherapy and to identify relevant influencing factors.

**Methods:** Patients who had achieved at least a 2-year seizure remission and started the withdrawal of antiseizure medication (ASM) were enrolled in this study. All patients were followed for at least 3 years or until seizure relapse. According to the number of ASMs at the time of withdrawalwas about twice than that, patients were divided into two groups: monotherapy group and polytherapy group. The Cox proportional hazards model was used to compare the recurrence risk of the two groups. Univariate analysis and multiple logistic regression analysis were used to analyze potential confounding variables between patients treated with monotherapy and polytherapy.

**Results:** A total of 188 patients (119 males and 69 females) were included. The average prescribed daily dose of most ASMs at the time of withdrawal was moderate or low (30–50% defined daily dose). The recurrence of most patients (89.2%) occurred within the first 3 years after withdrawal. The recurrence risk in patients treated with polytherapy at the time of withdrawal was about twice than that of the monotherapy group [*p* = 0.001, hazard ratio (HR) = 2.152, 95% confidence interval (CI) = 1.350–3.428]. Multivariate analysis showed that patients treated with polytherapy were significantly older at seizure onset [*p* = 0.024, odd ratio (OR) = 1.027, 95% CI = 1.004–1.052] and had a significantly longer duration of epilepsy before treatment (*p* = 0.004, OR = 1.009, 95% CI = 1.003–1.015) compared to patients in the monotherapy group. In addition, a history of perinatal injury was found to be an independent risk factor of seizure relapse in patients with ASM withdrawal.

**Conclusion:** The average prescribed daily dose of most ASMs at the time of withdrawal was moderate or low. Patients who received polytherapy at the time of withdrawal, particularly those with later seizure onset age and longer epilepsy duration before treatment, had a higher recurrence risk after ASMs withdrawal compared to patients treated with monotherapy.

## Introduction

Epilepsy is one of the most common chronic neurological diseases, affecting about 50 million people globally of all ages (1). Approximately 60–70% of patients become seizure-free by taking oral antiseizure medications (ASMs) (2). The continued use of ASM may have some adverse effects, such as drug reactions, impaired brain development (3), and increased psychological and economic burdens (4, 5), and remaining on ASM may not fully protect patients from seizure recurrence (6). The ideal goal of treating patients with epilepsy is complete seizure control and withdrawal of medical treatment without seizure recurrence. However, the rate of seizure relapse after ASM withdrawal ranges from 10 to 70% in different study designs and populations (7). It has been reported that resuming medication in 19% of patients [95% confidence interval (CI) = 15–24%] who relapse after withdrawal does not control the epilepsy as before, and 7–23% of patients develop chronic drug-resistant epilepsy (8). Therefore, it is necessary to perform early assessments of factors related to high recurrence risk to guide ASM withdrawal.

The development of new ASMs and improved an understanding of drug interactions support the potential application of polytherapy in epilepsy (9, 10). However, the results on the efficacy of monotherapy and polytherapy in patients with uncontrolled epilepsy are contradictory (11–13). Similarly, there is no consistent conclusion on the effect of monotherapy and polytherapy on seizure relapse after withdrawal in seizure-free patients. In 1984 (14), the Medical Research Council Antiepileptic Drug Withdrawal Study Group conducted a randomized controlled study of 1,013 patients who had been seizure-free for at least 2 years. The results showed that two or more ASMs were significantly associated with the recurrence (RR = 1.79, 95% CI = 1.34–2.39, *p* < 0.05). In 2013 (15), the Italian League Against Epilepsy report that eight of 23 studies showed a higher relapse risk in patients treated with polytherapy compared to monotherapy, but only two studies found significant differences in the multivariate analysis. In 2017 (16), a large-sample meta-analysis suggested that the number of ASMs was not an independent risk factor of seizure recurrence after withdrawal. With the advance in the development of new ASMs, it is worthy exploring whether the relationship between the number of ASMs and relapse after withdrawal has been changed. It has also been reported that the number of ASMs was significantly related to the mental state and quality of life of patients with epilepsy (17). Therefore, we conducted a retrospective observational study to compare the risk and timing of seizure relapse in seizure-free patients who took monotherapy or polytherapy after withdrawal and analyzed the clinical factors associated with seizure relapse.

## Materials and Methods

### Patient Enrollment

Patients who were diagnosed with epilepsy and had achieved at least 2 consecutive years of seizure control and started ASM withdrawal between September 1, 2008 and August 30, 2019 at the Renmin Hospital of Wuhan University (Wuhan, China) were enrolled in this study. All patients were followed for at least 3 years or until seizure relapse and had detailed medical records. The exclusion criteria were: patients with an acute symptomatic seizure; patients with juvenile myoclonic epilepsy; patients with progressive encephalopathy such as brain tumors; patients with a history of epilepsy resection surgery; and poor compliance (failed to take medicine as prescribed during the period of ASM withdrawal, such as medication omission and self-tapering) or incomplete medical records.

This study was approved by the Ethics Review Committee of Wuhan University Renmin Hospital. All participants provided informed consent.

### Data Collection

The following information were collected for analysis: sex, age at seizure onset, seizure type, etiology of epilepsy, family history of epilepsy, history of febrile seizures, perinatal injury, and craniocerebral injury, craniocerebral imaging results (magnetic resonance imaging and computed tomography), electroencephalogram (EEG) results before treatment and before ASM withdrawal, duration of epilepsy before medical treatment, the number and the dose of ASMs at the time of withdrawal, and the seizure-free interval before withdrawal.

The classification of seizure types and the definition of epilepsy etiology were based on the latest proposal of the International League Against Epilepsy (18). The classification of epilepsy syndrome was not included in this study because of the limited predictive value of epilepsy syndrome for success or failure after ASM withdrawal (19). In the present study, the etiology of epilepsy was divided into clear etiology (including structural, genetic, and infectious etiology) and unknown etiology. There were no patients with metabolic etiology or immune etiology due to the low incidence of metabolic epilepsy, the lack of genetic examination, and the poor prognosis of autoimmune-associated epilepsy (20, 21). The duration of epilepsy before treatment was defined as the time from seizure onset to registration at outpatient clinics. An abnormal EEG was defined as a specific focal, generalized epileptiform, or slow-wave abnormality.

### Study Design

Patients were divided into two groups according to the number of ASMs at the time of drug withdrawal: monotherapy group and polytherapy group. After ASM withdrawal, patients who remained seizure-free until the end of the study were defined as having seizure remission, while those who experienced seizure recurrence during the follow-up period were defined as having seizure relapse. ASM withdrawal was performed following the principle of gradual reduction, with one-quarter dose reduction every 3 months in the majority of patients. For patients treated with polytherapy, the first ASM was initially reduced and the withdrawal of the second drug started after the complete reduction of the former one. The follow-up period starts with the reduction of the first ASM. The primary outcome of this study was the comparison of the recurrence risk after ASM withdrawal between the monotherapy and polytherapy groups. Factors that were related to the recurrence risk of the two groups were also explored.

### Sample Size Calculation

The sample size was calculated according to previous data of patients with ASMs withdrawal (14, 22–24). In real-world clinical practice, the proportion of patients in the two groups was not evenly distributed at the time of ASM withdrawal. Base on the data of previous studies, the sample size ratio of the polytherapy group to the monotherapy group was set as 0.3. The recurrence rates of the polytherapy to the monotherapy were set as 0.7 and 0.4, respectively. In addition, the alpha value was 0.05 and the power was 0.90. The analysis using the PASS 15.0 software showed that the sample size of the polytherapy group was 35 and that of the monotherapy group was 118. Therefore, the total number of patients should be 153. In this study, we included as many eligible patients as possible to ensure the reliability and validity of the data.

### Statistical Analysis

Statistical analysis was performed using SPSS software 25.0 package (SPSS Inc., IL, USA). In univariate analysis, Mann–Whitney-test and chi-squared-test were used to compare continuous and categorical variables, respectively. Variables found to be statistically significant between the two groups on univariate analysis were further investigated using multivariate logistic regression analysis. Multivariate Cox regression analysis was performed to compare the relapse risk between the two groups after ASM withdrawal, adjusted for the effects of potential confounding factors. Firstly, multivariate Cox stepwise regression analysis was used to examine other confounding factors that unbalanced variables between the two groups and significantly affected relapse. Next, variables that significantly affected seizure relapse were included in the adjusted Cox proportional hazards model. The number of ASMs was the only covariate in the unadjusted Cox model. Kaplan–Meier survival analysis was used to determine the cumulative relapse rate of the two groups after ASM withdrawal. All statistical tests were two-tailed, and *p* < 0.05 was considered statistically significant.

## Results

### Demographic and Clinical Characteristics at Baseline

A total of 188 patients (119 males and 69 females) who met the inclusion criteria were recruited in this study. The mean duration of follow-up was 45.3 months (±32.5, range = 0.5–135). The average seizure-free interval before the initiation of ASM withdrawal was 3.2 years (±0.5, range = 2.0–6.2), and the majority of ASM withdrawal (91%) occurred in the third seizure-free year. Among all patients, 135 (71.8%) received monotherapy and 53 (28.2%) received polytherapy. The demographic and clinical characteristics of the two groups at baseline are shown in Table 1.

**Table 1:** Univariate analysis of the demographic and clinical characteristics of patients treated with monotherapy and polytherapy.

**Variables**	**Monotherapy, *N* = 135**	**Polytherapy, *N* = 53**	***p-*value**
Age at seizure onset			0.041
Median (years)	13.0	16.0	
Mean (years)	17.0	21.5	
Duration of epilepsy before treatment			0.035
Median (months)	8.0	24.0	
Mean (months)	26.0	51.2	
Gender			0.246
Men	82 (60.7%)	37 (69.8%)	
Women	53 (39.3%)	16 (30.2%)	
Seizure type			0.192
Focal seizure	81 (60.0%)	25 (47.2%)	
Generalized seizure	42 (31.1%)	24 (45.3%)	
Unknown seizure	12 (8.9%)	4 (7.5%)	
Etiology of epilepsy			0.818
Clear etiology	46 (34.1%)	19 (35.8%)	
Unknown etiology	89 (65.9%)	34 (64.2%)	
Perinatal injury			0.848
No	121 (89.6%)	48 (90.6%)	
Yes	14 (10.4%)	5 (9.4%)	
History of febrile seizure			0.507
No	128 (94.8%)	49 (92.5%)	
Yes	7 (5.2%)	4 (7.5%)	
Family history of epilepsy			0.713
No	129 (95.6%)	50 (94.3%)	
Yes	6 (4.4%)	3 (5.7%)	
History of craniocerebral injury history			0.303
No	104 (77.0%)	37 (69.8%)	
Yes	31 (23.0%)	16 (30.2%)	
EEG results before medicine treatment			0.073
Normal	23 (17.0%)	10 (18.9%)	
Abnormal	96 (71.1%)	30 (56.6%)	
Unknown	16 (11.9%)	13 (24.5%)	
EEG results before withdrawal			0.370
Normal	57 (42.2%)	23 (43.4%)	
Abnormal	43 (31.9%)	12 (22.6%)	
Unknown	35 (25.9%)	18 (34.0%)	
MRI or CT result			0.332
Normal	71 (52.6%)	27 (50.9%)	
Abnormal	31 (23.0%)	17 (32.1%)	
Unknown	33 (24.4%)	9 (17.0%)	

*EEG, electroencephalogram; MRI, magnetic resonance imaging; CT, computed tomography*.

In the monotherapy group, oxcarbazepine (38/135, 28.1%) was the most common drug, followed by valproate (29/135, 21.5%), lamotrigine (26/135, 19.3%), levetiracetam (24/135, 17.8%), carbamazepine (14/135, 10.4%), and topiramate (4/135, 3.0%). In the polytherapy cohort, all patients received a combination of two ASMs. The most common combination was valproate and lamotrigine (24/53, 45.3%), followed by valproate and oxcarbazepine (8/53, 15.1%), levetiracetam and lamotrigine (7/53, 13.2%), and valproate and carbamazepine (5/53, 9.4%). As defined by the World Health Organization, the defined daily dose (DDD) is the assumed average maintenance dose per day for its main indication in adults (25). The mean prescribed daily dose (PDD) and the percentage of DDD (PDD/DDD) in the two groups are shown in Table 2.

**Table 2:** DDD, mean PDD, and percentage of DDD in the two groups.

**Antiseizure medication**	**DDD (mg)**	**Monotherapy**		**Polytherapy**	
		**Mean PDD (mg)**	**DDD%**	**Mean PDD (mg)**	**DDD%**
VPA	1,500	585.86	39.06	736.49	49.10
CBZ	1,000	364.29	36.43	400.00	40.00
OXC	1,000	536.84	53.68	681.25	68.13
LTG	300	97.12	32.37	99.26	33.09
LEV	1,500	473.96	31.60	920.45	61.37
TPM	300	112.50	37.50	100.00	33.33

*DDD, defined daily dose; PDD, prescribed daily dose; VPA, valproate; CBZ, carbamazepine; OXC, oxcarbazepine; LTG, lamotrigine; LEV, levetiracetam; TPM, topiramate*.

### Comparison of Seizure Relapse Between the Monotherapy and Polytherapy Groups

The overall relapse rate was 39.4% (74/188) at the end of the study. Among all patients, 32.6% (44/135) and 56.6% (30/53) experienced seizure relapse in the monotherapy and polytherapy groups, respectively. According to the multivariate Cox stepwise regression analysis, the history of perinatal injury was the only confounding factor that affected recurrence (*p* < 0.05; Table 3). The recurrence risk of patients treated with polytherapy was significantly higher than that of the monotherapy group [hazard ratio (HR) = 2.152, 95% CI = 1.350–3.428] after adjusting for the history of perinatal injury (Table 4).

**Table 3:** Effect of various factors on seizure relapse: multivariate Cox regression analysis.

**Variables**	**HR**	**95% CI**	***p*-value**
Number of ASMs	1.958	1.188–3.229	0.008
Age at seizure onset	1.008	0.989–1.028	0.403
Gender	1.005	0.611–1.655	0.984
Seizure type	0.989	0.974–1.003	0.117
Etiology of epilepsy	1.206	0.671–2.168	0.530
Perinatal injury	2.549	1.294–5.012	0.007
History of febrile seizure	0.347	0.080–1.511	0.158
Family history of epilepsy	2.134	0.878–5.188	0.094
History of craniocerebral injury	1.685	0.952–2.983	0.073
EEG results before medicine treatment	0.920	0.604–1.401	0.698
EEG results before withdrawal	0.895	0.667–1.201	0.460
MRI or CT result	0.764	0.537–1.086	0.134
Duration of epilepsy before treatment	1.003	0.999–1.007	0.111
Seizure-free interval	0.955	0.436–2.096	0.910

*HR, hazard ratio; CI, confidence interval; ASM, antiseizure medication; EEG, electroencephalogram; MRI, magnetic resonance imaging; CT, computed tomography*.

**Table 4:** Comparison of seizure relapse risk between patients treated with monotherapy and polytherapy.

	**Unadjusted monotherapy**			**Adjusted**^a^ **monotherapy**		
	***p***	**HR**	**95% CI**	***p***	**HR**	**95% CI**
Polytherapy	0.002	2.102	1.320–3.347	0.001	2.152	1.350–3.428

*HR, hazard ratio; CI, confidence interval*.

a *Adjusted for the history of perinatal injury in the multivariate Cox regression analysis*.

**Figure 1:**
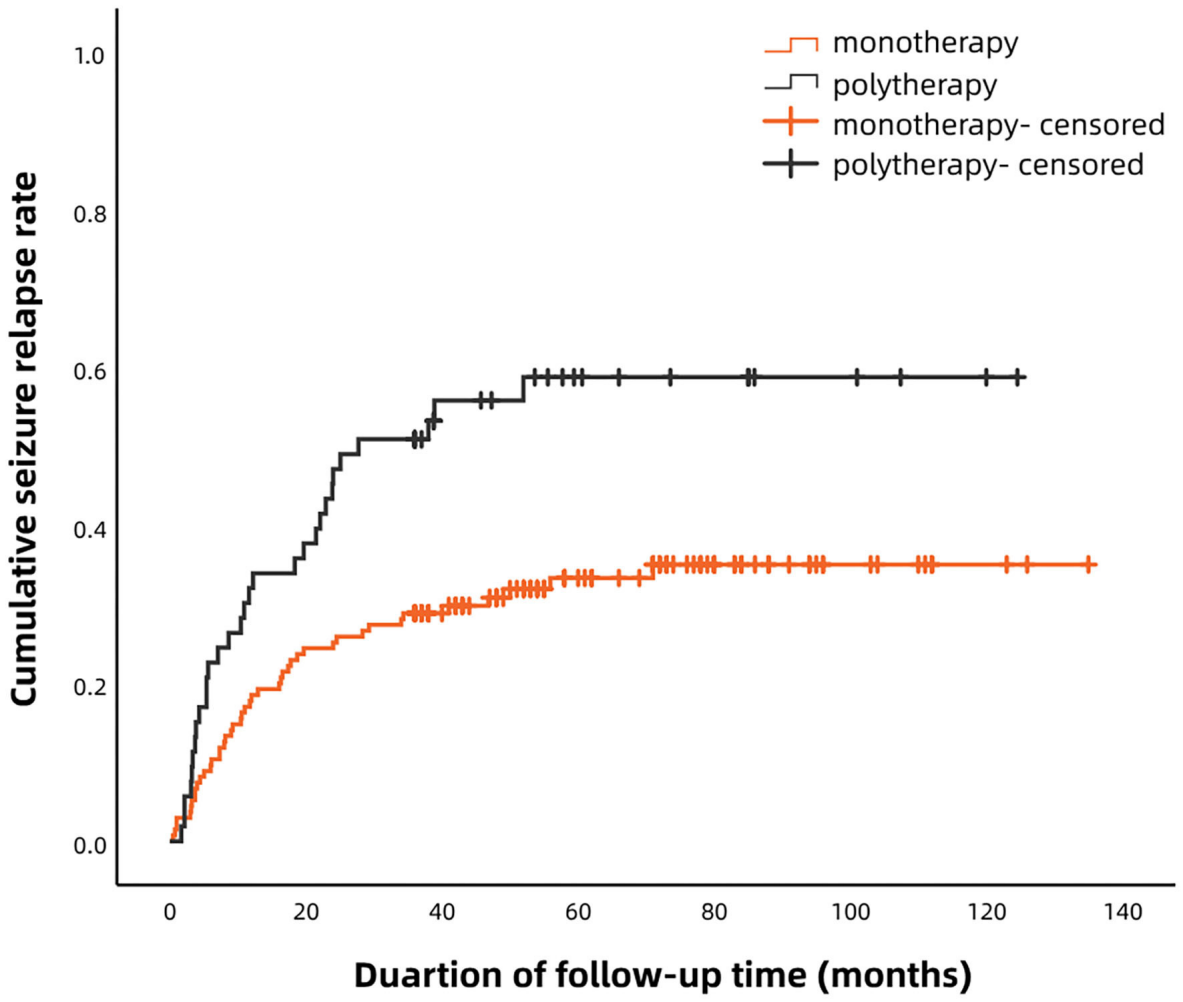
Kaplan–Meier analysis of the cumulative recurrence rates in patients treated with monotherapy and polytherapy.

**Figure 2:**
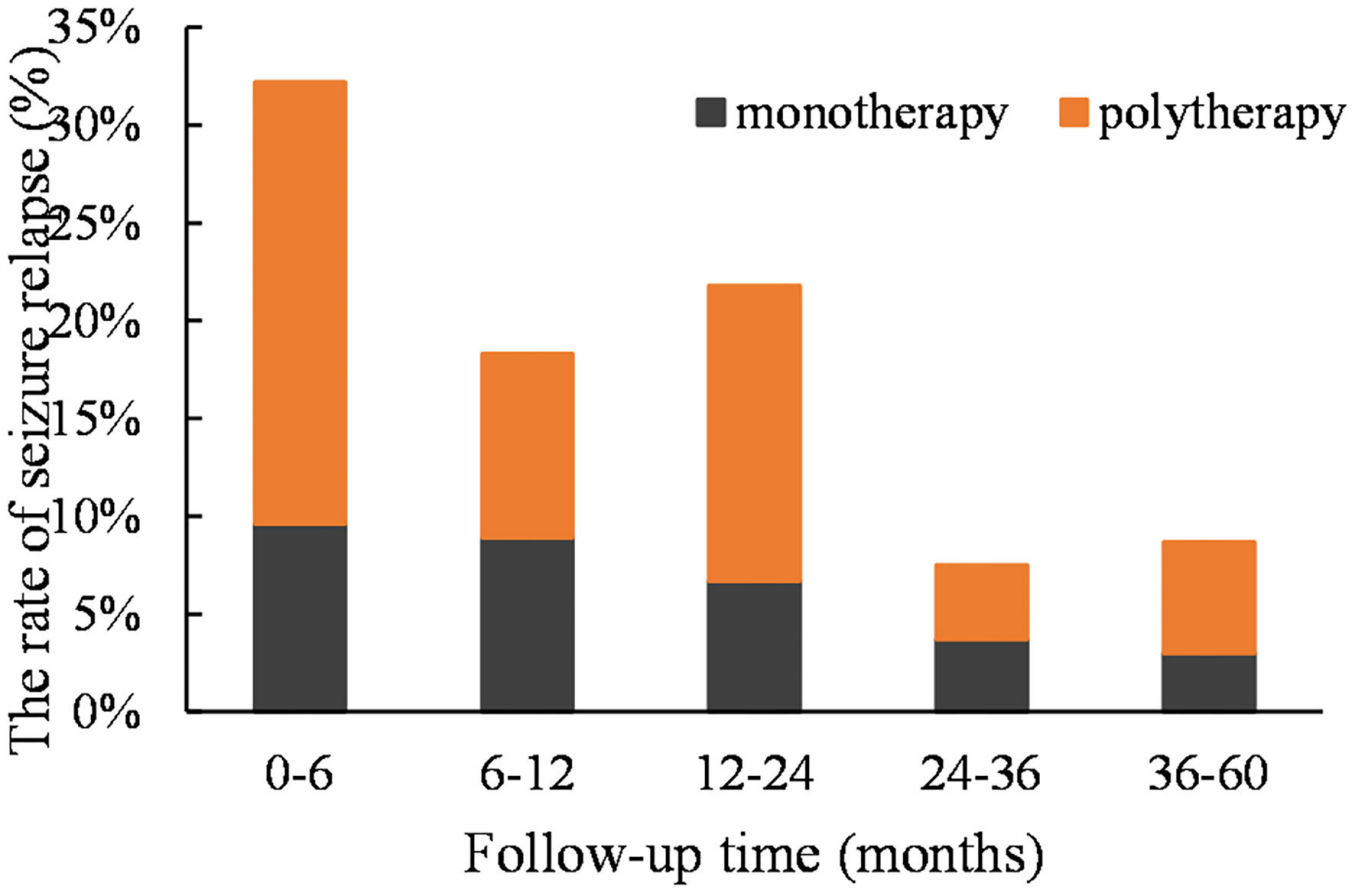
The staged recurrence rates of two groups of patients at 0–6, 6–12, 12–24, 24–36, and 36–60 months. The peak occurred in the first 12 months (mainly in the first 6 months) in both groups.

According to the Kaplan–Meier survival analysis, the cumulative recurrence rates in the monotherapy group at 6, 12, 24, 36, and 60 months were 9.6, 18.5, 25.2, 28.9, and 31.8%, respectively; these were 22.6, 32.1, 47.2, 50.9, and 56.6%, correspondingly, in the polytherapy group (Figure 1). The staged recurrence rates of patients in the two groups at 0–6, 6–12, 12–24, 24–36, and 36–60 months are shown in Figure 2. In the monotherapy group, the recurrence rates were 9.6, 8.9, 6.7, 3.7, and 3.0%, respectively, whereas the rates in the polytherapy group were 22.6, 9.4, 15.1, 3.8, and 5.7%, respectively. Chi-squared-test showed that the risk of early relapse (6 months) in the polytherapy group was significantly higher than that in the monotherapy group (22.6 vs. 9.6%, *p* = 0.018). Most recurrences (89.2%) occurred within 3 after withdrawal.

### Differences in Demographic and Clinical Characteristics Between Two Groups

Univariate analysis showed that patients treated with polytherapy had an older onset age (median = 16.0 vs. 13.0 years, mean = 21.5 vs. 17.0 years, *p* = 0.041) and a longer duration of epilepsy before treatment (median = 24.0 vs. 8.0 months, mean = 51.2 vs. 26.0 months, *p* = 0.035) compared to those treated with monotherapy. No significant differences were found between the two groups in sex, seizure type, etiology of epilepsy, family history of epilepsy, history of febrile seizures, perinatal injury, craniocerebral injury, EEG results before medicine treatment and before withdrawal, and craniocerebral imaging results (all *p* > 0.05; Table 1). In the multivariate logistic regression analysis, the age at seizure onset (*p* = 0.024, OR = 1.027, 95% CI = 1.004–1.052) and the duration before treatment (*p* = 0.004, OR = 1.009, 95% CI = 1.003–1.015) remained significantly different between the monotherapy and polytherapy groups (Table 5).

**Table 5:** Multivariate logistic regression analysis of the demographic and clinical variables between the monotherapy and polytherapy groups.

**Variables**	***p-*value**	**OR**	**95% CI**
Age at seizure onset	0.024	1.027	1.004–1.052
Duration of epilepsy before treatment	0.004	1.009	1.003–1.015

*OR, odds ratio; CI, confidence interval*.

## Discussion

In this study, all patients were followed for at least 3 years or until seizure relapse after the initiation of ASM withdrawal, which was considered sufficient to assess the overall prognosis of seizure-free patients by Park et al. (24). The total recurrence rate (39.4%) measured in our study was consistent with that reported in previous investigations (10–70%) (7). Consistent with previous studies (26–29), the average PDD of most ASMs at the time of withdrawal in our study was moderate or low (30–50% DDD), except for oxcarbazepine and levetiracetam (>60% DDD), which was slightly higher in the polytherapy group.

Several recent studies have investigated the relationship between seizure relapse and the number of ASMs in patients with ASM withdrawal, but there has been no consistent conclusion. Our results demonstrated that patients treated with polytherapy had a higher seizure relapse risk than those with monotherapy (HR = 2.152, 95% CI = 1.350–3.428), which was in line with previous studies (23, 30–32). We also found that the risk of early recurrence (within the first 6 months) in polytherapy-treated patients was significantly higher than that of the monotherapy group (22.6 vs. 9.6%, *p* = 0.018). Others, however, did not find that the number of ASMs was correlated with seizure relapse (33, 34). The Italian League Against Epilepsy recommends that patients who receive polytherapy be warned of a higher risk of recurrence, but the withdrawal of ASM could be considered, in particular when no other risk factors are present (15).

A recent review reported that the peak of relapse was in the first 12 months (mainly in the first 6 months) after withdrawal and tended to decrease thereafter (35), which was similar to our findings (in both monotherapy- and polytherapy-treated patients). Therefore, we concluded that the relapse trend was not correlated with the number of ASMs, in line with a previous study (24).

In the present study, we retrospectively analyzed the demographic and clinical characteristics of the two groups and found that patients treated with polytherapy had a significantly later age of onset (median = 16.0 vs. 13.0 years, mean = 21.5 vs. 17.0 years) and longer duration of epilepsy before medical treatment (median = 24.0 vs. 8.0 months, mean = 51.2 vs. 26.0 months) compared with the monotherapy group.

The effect of age at seizure onset on the recurrence of patients with ASM withdrawal has been extensively studied. An early meta-analysis of 25 studies has reported that patients with onset during adolescence (10–20 years) or adulthood (>20 years) have a higher risk of recurrence than those with onset during childhood (<12 years) (7). An individualized prediction model of seizure recurrence in seizure-free patients after withdrawal has shown that the risk of seizure relapse is high when the onset is at birth, drops to a nadir at the age of 3–4 years, starts to rise again until age 10 years, plateaus until age 25 years, and then rises further with advancing age of onset (16). Consequently, the age at first seizure onset is positively correlated with seizure relapse after ASM withdrawal. In addition, many studies have reported that the duration of epilepsy before treatment and the duration of active epilepsy were significantly correlated with seizure relapse after ASM withdrawal (23, 24, 34, 36). The duration of active epilepsy is related to the severity of the disease. Park et al. (24) reported that a longer duration of symptoms might be related to the intrinsic reactivation of epilepsy. In terms of pathogenesis (37), it has been reported that repeated seizures induce neuronal loss and mossy fiber sprouting in the hippocampus, which may reinforce the production of excitatory recurrent circuits and therefore aggravate epilepsy. Therefore, we conclude that a later age of seizure onset and a longer course of epilepsy before treatment were important characteristics of polytherapy-treated patients. We also speculate that these two factors may be related to disease severity and poor prognosis.

The correlation between EEGs and epilepsy recurrence is disputable. Previous evidence has demonstrated that an abnormal EEG before ASM withdrawal is a predictor of the relapse (7, 38). In this study, we did not observe significant differences in the EEG results collected before treatment and before withdrawal between the monotherapy and polytherapy groups, probably due to the relatively small sample size and the fact that EEG was only measured for 2 h. Yao et al. (39) reported that abnormal EEG during withdrawal was a risk factor for recurrence. Su et al. (33) found that abnormal EEG results within the first year after ASM withdrawal was an independent predictor of seizure relapse and suggested the EEG test during the first year after ASM withdrawal is highly recommended. Therefore, the detection of EEG during and after drug withdrawal is also worthy of attention. In recent years, the value of quantitative pharmaco-EEG in predicting and evaluating treatment response and side effects of ASM has started to gain scientific interest (40). Studies of patients with temporal lobe epilepsy have shown that quantitative EEG at both resting state and after treatment has a high predictive significance for the effect of drug treatment, which may offer new prognostic biomarkers for patients with epilepsy (41). Therefore, whether the application of quantitative pharmaco-EEG in seizure-free patients with ASM withdrawal can improve the predictive value of EEG warrants further investigation.

The discontinuation of medications for patients who achieved long-term seizure freedom has always been a concerning issue. According to the guidelines of the Italian League Against Epilepsy, the minimum period of seizure freedom is 2 years (15). In the present study, 171 patients were seizure-free for at least 3 years before withdrawal (eight patients: >4 years; two patients: >5 years). However, Camfield and Camfield (19) suggested that children who have been seizure-free for 1–2 years may consider the discontinuation of ASM treatment and a longer seizure-free period has no significant effect on the success rate, whereas adult patients should remain seizure-free for at least 4 years before ASM withdrawal. In addition, a prospective controlled study by Wang et al. (42) found that a seizure-free period of more than 5 years before withdrawal significantly reduced the relapse risk compared with a seizure-free period of 2–3 years for adults with focal epilepsy. Recently, a large-sample meta-analysis of seizure recurrence after ASM withdrawal supplemented that every added seizure-free year reduces the risk of recurrence (16). Therefore, it may be more appropriate to extend the seizure-free interval for patients with a high risk of recurrence.

According to previous reports, factors such as gender, seizure type, etiology of epilepsy, EEG, imaging examination, history of perinatal injury and brain injury, and family history of epilepsy are related to seizure recurrence after withdrawal. In the current study, however, no differences were observed in these factors between the monotherapy and polytherapy groups. On one hand, recall bias might occur when patients are asked to provide an overview of their medical history. On the other hand, the population and study design may differ among studies.

This study had several limitations. Firstly, as an observational study, the mechanism underlying the association between polytherapy and relapse was not explored. Secondly, there was no randomization and no control group due to the nature of the retrospective study. Finally, due to the relatively small sample size, some prognostic factors may be missed. Multicenter, large-scale, prospective controlled studies are needed to further investigate the long-term recurrence of patients treated with monotherapy and polytherapy.

## Conclusions

Our study suggests that clinicians consider extending the seizure-free interval of patients who have achieved epilepsy remission by polytherapy, especially those with a later age of onset and a longer course before treatment.

## Data Availability Statement

The original contributions generated for the study are included in the article/supplementary material, further inquiries can be directed to the corresponding author.

## Ethics Statement

The studies involving human participants were reviewed and approved by Ethics Review Committee of Wuhan University Renmin Hospital. Written informed consent from the participants' legal guardian/next of kin was not required to participate in this study in accordance with the national legislation and the institutional requirements.

## Author Contributions

YW designed and drafted the study. LX collected and analyzed the data. RL analyzed the data. YL and JL were responsible for patient follow-up. QZ edited the pictures. SP supervised the study. All the authors read and approved the final manuscript.

## Conflict of Interest

The authors declare that the research was conducted in the absence of any commercial or financial relationships that could be construed as a potential conflict of interest.
